# Automatic assessment of infant carrying and holding using at-home wearable recordings

**DOI:** 10.1038/s41598-024-54536-5

**Published:** 2024-02-28

**Authors:** Manu Airaksinen, Einari Vaaras, Leena Haataja, Okko Räsänen, Sampsa Vanhatalo

**Affiliations:** 1 BABA Center, Pediatric Research Center, Department of Clinical Neurophysiology, New Children’s Hospital and HUS Imaging, Helsinki University Hospital, Helsinki, Finland; 2 Department of Physiology, University of Helsinki, Biomedicum 1, Room B129b, Haartmaninkatu 8, 00290 Helsinki, Finland; 3 Unit of Computing Sciences, Tampere University, P.O. Box 553, 33101 Tampere, Finland; 4 Department of Pediatric Neurology, Children’s Hospital, Helsinki University Hospital and University of Helsinki, Helsinki, Finland

**Keywords:** MAIJU, Biomedical engineering, Paediatric research

## Abstract

Assessing infant carrying and holding (C/H), or physical infant-caregiver interaction, is important for a wide range of contexts in development research. An automated detection and quantification of infant C/H is particularly needed in long term at-home studies where development of infants’ neurobehavior is measured using wearable devices. Here, we first developed a phenomenological categorization for physical infant-caregiver interactions to support five different definitions of C/H behaviors. Then, we trained and assessed deep learning-based classifiers for their automatic detection from multi-sensor wearable recordings that were originally used for mobile assessment of infants’ motor development. Our results show that an automated C/H detection is feasible at few-second temporal accuracy. With the best C/H definition, the automated detector shows 96% accuracy and 0.56 kappa, which is slightly less than the video-based inter-rater agreement between trained human experts (98% accuracy, 0.77 kappa). The classifier performance varies with C/H definition reflecting the extent to which infants’ movements are present in each C/H variant. A systematic benchmarking experiment shows that the widely used actigraphy-based method ignores the normally occurring C/H behaviors. Finally, we show proof-of-concept for the utility of the novel classifier in studying C/H behavior across infant development. Particularly, we show that matching the C/H detections to individuals’ gross motor ability discloses novel insights to infant-parent interaction.

## Introduction

Assessment of infants’ daily activities and spontaneous behaviors has become an important target in a variety of disciplines, including developmental psychology, early education, social work, as well as many fields of health and medical care^1–4^. These assessments are conventionally based on a large repertoire of questionnaires for parents that can be complemented with direct observations by study personnel in the lab or home environment. However, the parental questionnaires are unavoidably subjective and only partially reliable, while the direct observations by study personnel are subjective, resource-intensive, and they interfere with a child's natural behavior in one way or another. Novel methods are thus needed to obtain objective, quantitative, and reliable measures of a child's typical activity^3,5,6^.

A key challenge in infant studies is to collect ecologically relevant data that typically implies out-of-hospital/lab recordings, i.e., measurements done at home or in home-like environments. Recent technological development has introduced new possibilities for recording and analyzing such out-of-hospital recordings based on measuring movements, audio, or video^1,7^. Parental surveys have indicated privacy concerns with in-home audio and video recordings^8,9^ favoring the use of movement sensors that collect far less identifiable data. Such movement sensors would typically collect tri-axial measures of both linear acceleration (accelerometer) and angular velocity (gyroscope)^10,11^, and they can be attached to infants individually^12,13^ or by using more comprehensive multi-sensor suits^5,10,14^. Analysis of the data is traditionally based on straightforward quantification of the amount of movements (i.e., an actigraphy-type measure)^15^, while machine learning-based analysis enables more sophisticated and higher level interpretations of the movement data^10,16^.

Performing research out-of-hospital/lab implies data collection in partly or completely unsupervised settings, which poses several additional challenges that are not encountered when recordings are performed by professionals in a fully controlled environment. In studies on infants' spontaneous activity, it is particularly important to distinguish time periods where the infant has been moving him/herself vs. times where the infant was carried or held by someone^17^. In addition to being a potential confounder in movement assessment, parental holding behavior is an interesting measure *per se*^18^ since it can impact infants’ developmental outcomes^19^. Detection of infants’ carrying/holding is often attempted by applying movement thresholds^15,16^ assuming that externally generated movements are larger than infant-generated movements. An alternative strategy is to measure co-incident activities in sensors attached to both the infant and the caregiver^13^, relying on the assumption that carrying/holding is reflected in the movements of both the infant and the parent synchronously. While the studies may report relatively high classifier performances with the given training datasets, there are as of yet insufficient means to measure holding/carrying behavior in unsupervised settings, such as out-of-hospital/lab without direct guidance by the research personnel.

Here, we set out to design and construct a deep learning-based classifier for the multi-sensor recordings with the wearable MAIJU (Motor Assessment of Infants with a Jumpsuit^10^). We tested and trained the algorithm systematically in different kinds of spontaneous holding and carrying behaviors recorded from spontaneous every-day activity. Carrying and holding the infant by the adult caregiver is not a stereotyped movement behavior akin to crawling or walking; it is rather a spectrum of behaviors with varying levels of adult-infant physical contacts or varying effects on infant’s own movements. Such non-discrete behavioral spectra also pose challenges to human visual classification^10,20,21^, the basis of supervised algorithmic training; we therefore developed an intuitive classification scheme and tested its related human inter-rater agreement levels to provide a benchmark for the classifier performance^10,22^. We also examined the effect of category combinations on the classifier performance. Finally, we provide a proof-of-concept use of the newly trained classifiers in assessing unsupervised multi-hour home recordings in infants at different ages, and we show how the carrying/holding classifier can be matched with infants’ motor performance during the study sessions.

## Methods

### Overview

**Figure 1:**
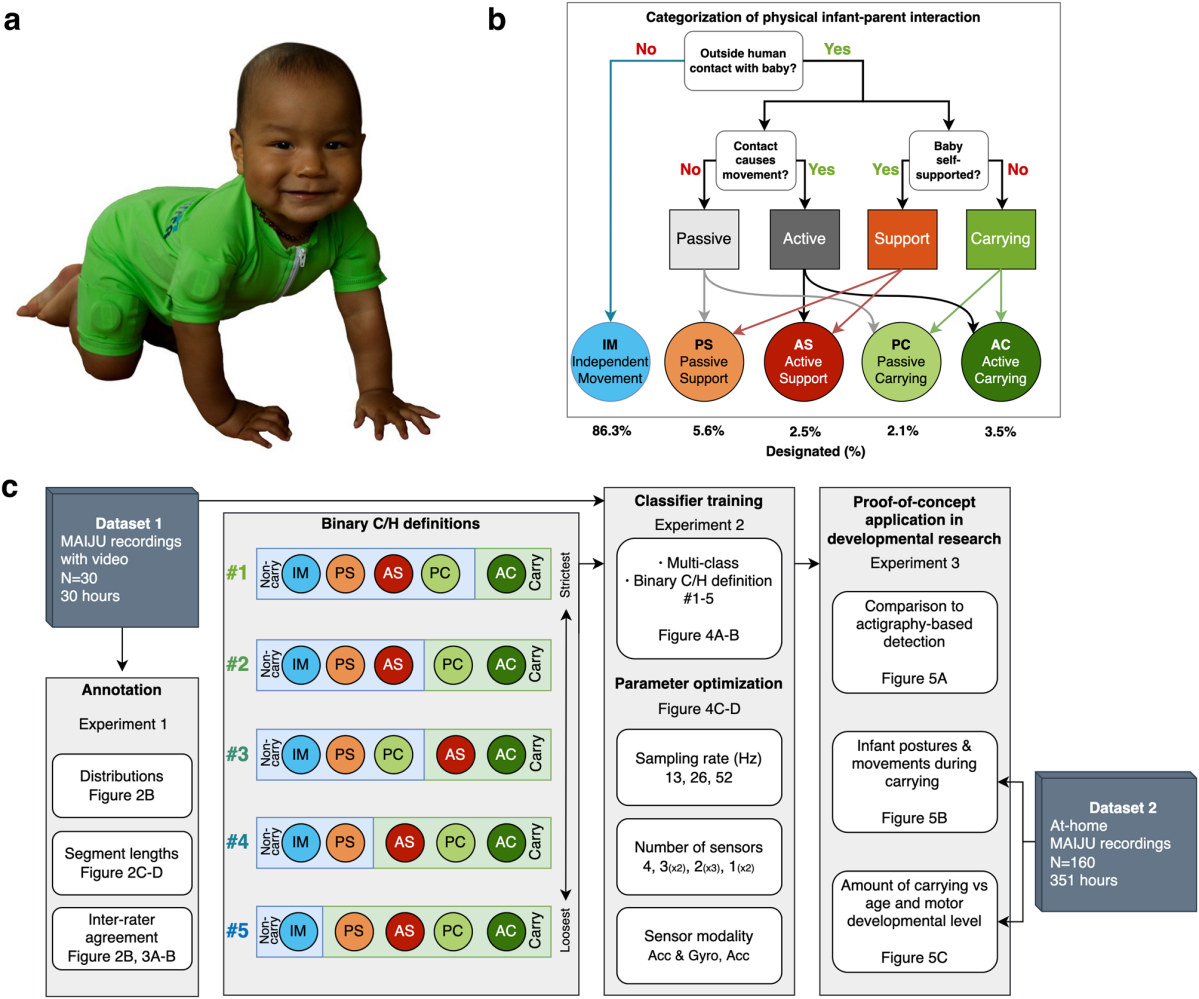
(**a**) Photograph of a 10-month-old infant with the MAIJU wearable. (**b**) Decision tree diagram used for the annotation of physical infant-parent interactions. (**c**) Block diagram of the full study design. The middle box shows the five carrying definitions (#1–5) based on the annotated categories shown in B. Image of the infant is reproduced with written parental consent. For further details, see Table 1 and the paragraph on the rationale of the categorization scheme.

The overall rationale in our present development study is presented in Fig. 1. The recordings were performed with the MAIJU wearable (Fig. 1a), and the classifier training was performed using annotations that were available from synchronized video recordings. All videos were annotated independently by two observers using the categorization scheme for physical infant-parent interaction (Fig. 1b); these annotations were then combined into five different binary definitions of infant carrying and/or holding (#1–5; Fig. 1c), jointly called carrying/holding detection (C/H). The suitability of these carrying definitions was tested first with human inter-rater agreement, then by assessing performance of automated classifier algorithms. The best-performing binary definition was investigated further to examine how the classification performance would be affected by the following changes in the recording setup: (1) sensor placement or number of sensors, (2) recording sampling rate, and (3) sensor modality (accelerometer vs gyroscope). Finally, we conducted proof-of-concept experiments to showcase the potential utility of IMU sensor-based recordings in the study of infant carrying.

### MAIJU recordings

**Figure 2:**
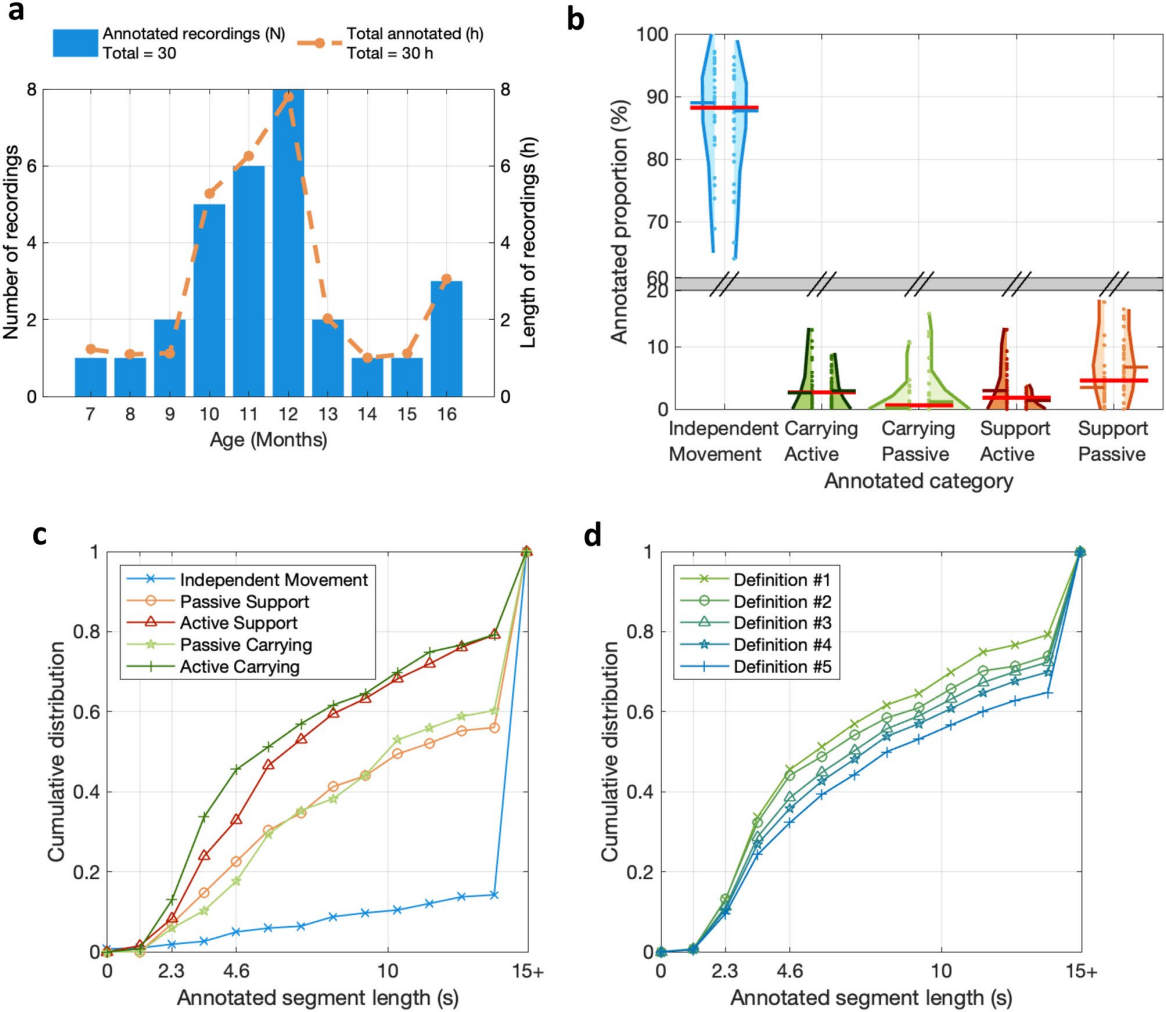
Summary of the recording dataset and annotation statistics. (**a**) Age-based histograms for the number of infants (blue) and the amount of annotated data (orange). (**b**) Proportions of annotated data in each recording (dots) and each annotator (left and right-side violin plots). (**c**) Cumulative distributions of the segment lengths of different annotations, i.e., categories of physical infant-caregiver interactions. Note the predominance of brief segments that characterize the infant-caregiver interactions, as compared to the very long segments that are typically present during independent movement. (**d**) Cumulative distributions of the segment lengths for different carrying definitions. Note that most of carrying/holding by any definition is brief, exhibiting durations less than 10 s.

We used data from a novel multi-sensor infant wearable MAIJU (Motor ability Assessment of Infants with a JUmpsuit; Fig. 1a)^10^. It is essentially a commonplace full body overall swimsuit equipped with four movement sensors, one in each limb with a proximal placement. The sensors stream inertial measurement unit (IMU) data (3-axis accelerometer and gyroscope) over a low-energy Bluetooth (BLE, v5.0) connection to a nearby mobile phone using a custom-built iOS application, “MAIJU logger” (Kaasa GmbH, Düsseldorf, Germany). The data is collected at 52 Hz or 1248 measurement values per second (for further details, see^10^). As described in detail in^10^, the non-videoed in-home recordings were mostly performed by the parents without direct supervision. The present study collated hour-long recordings that were performed in the infants’ homes during free play time^10^. The dataset (DS-1) used for classifier training and performance testing included synchronized video recordings to allow human annotations. DS-1 consisted of *N* = 30 infants between 6.6 and 16.8 months of age with recordings lasting from 57 to 62 min (average 60 min), and a total recording time corresponding to 1799 min (Fig. 2a–d). Another dataset, DS-2, was used for the proof-of-concept assessment and it included minimally controlled at-home recordings of *N* = 160 infants between 4.1 and 18.4 months of age, consisting of parent-reported “free play”. These recordings lasted from 22 to 393 min (average 131 min), with a total recording time corresponding to 21,082 min (351 h). Both datasets (DS-1 and 2) aim to utilize data collected during “free play” of the infants. During “free play” the parents were instructed to not spontaneously initiate C/H behavior but were encouraged to respond naturally to the infants’ initiations/behavior (i.e., infant requiring attention or consolation, or safe-guarding from harm’s way). In DS-2, the parents were instructed to keep a record of the rough time-windows during which the “free play” occurred. Both datasets were obtained as convenience samples from typically developing infants whose parents were willing to participate in the study.

The recordings in DS-2 were processed with the MAIJU analysis pipeline introduced in^10^ where the recordings are automatically classified for parallel posture (7 categories) and movement (9 categories) tracks at a 1-s level time resolution. The posture and movement categories were collated into recording-specific distributions that were used as feature vectors in computing the “motility age” of the infant based on the recording, denoted as BIMS (BABA Infant Motor Score)^10,23^. In BIMS, Gaussian Process Regression (GPR) is used to regress (using cross-validation) the recording-based feature vectors against the chronological ages at recording. The resulting age estimates are normalized into a range of 0–100, where 0 denotes undeveloped gross motor capacity (ca. 4-month-old level), and 100 denotes the full acquisition of fluent walking (ca. 16-month-old level).

### Description of the C/H categorization scheme

The behavioral repertoire of physical caregiver-infant interactions is large, and it can be categorized in any number of complementary dimensions. For instance, one could categorize it according to the assumed intention of the interaction (e.g., “parent is soothing the baby”), or more objectively by describing the mere physical motions that can be observed in the infant during the intervention (e.g., “the baby is being held with a rocking motion”). The overall motivation in our phenomenological categories is to define what is the relationship of the child (wearing the sensors) to the outside source of physical interaction (the caregiver). Obviously, the IMU-based movement sensors are good for sensing physical attributes of the infant-caregiver interaction (acceleration, angular velocity), but they cannot sense the human intentions present in the interactions. The IMU sensors should therefore not be expected to differentiate between infant-caregiver interactions where the caregiver is not physically moving. The proposed categorization scheme thus describes the physical movements only; notably, this could still allow ad-hoc inferences about intentionality of the actions if needed.

### Rationale of the categorization scheme

As described in Fig. 1 and Table 1, the proposed categorization proceeds through a simple and intuitive binary decision tree: The first stage question (*Is the infant in physical contact with another human?*) determines whether the infant is moving independently. The second stage presents two questions (*Is the infant self-supported?* and *Is the contact moving the infant?*) that jointly determine the category of physical caregiver-infant interaction, which produces four alternatives: The interaction is considered as *Passive Support (PS)* if there is only touch or support without physically displacing the infant (e.g., child crawling on or leaning towards an adult), while the interaction is called *Active Support (AS)* if the infant is also being moved (e.g., walking the infant from the hand). Alternatively, the interaction is considered *Passive Carrying (PC)* if the adult is holding the infant without moving (e.g., laying still on the lap), while *Active Carrying (AC)* refers to a situation where infant is being moved while carrying (e.g., lifting from the floor or carrying around).

**Table 1:** Proposed physical infant-caregiver interaction categorization scheme, alongside the human inter-rater agreement (kappa).

Activity	Definition	Kappa
Independent movement (IM)	There is no outside contact with the infant present	.85
Passive support (PS)	Infant is in physical contact with another person. Infant is primarily self-supported (i.e., maintain postural balance by themself). Contact does not cause movement	.46
Active support (AS)	Same as PS but contact causes movement	.32
Passive carry (PC)	Infant is in physical contact with another person. The contact primarily maintains the infant’s postural balance. Contact does not cause movement	.45
Active carry (AC)	Same as PC but contact causes movement	.75
Overall		.67

Please see further description in the paragraph “Rationale of the categorization scheme” and Fig. 1.

It is obvious that all these behavioral categories could be readily subdivided to many sub-categories. Carrying could be divided to, for instance, holding, lifting, and laying; Likewise, support could be divided to at least postural support, movement support, and touching. Such detailed behavioral descriptions would, however, complicate the taxonomy too much to allow an appropriate training of classifier algorithms with any reasonably achievable datasets. They would directly cause at least perceptual ambiguity in the definitions. For instance, the concept “self” in the support class could be challenged by comparing infant’s sitting on the caregiver’s lap versus in a chair. From the infant's perspective, they could be physically comparable situations, while from the human interaction perspective the parent could be considered to carry rather than support. In the proposed categorization scheme, this example would fall into the “support” category if the baby is sitting on the lap with their back straight (contact only from the legs), but into “carrying”, if they are leaning for support from the parent. This detail in our definitions differs slightly from that reported by^13^: Their differentiation between “holding” vs “non-holding” was based on observing whether the “child’s weight is completely supported by caregiver” [sic] or not; hence, the example with sitting on the lap would be categorized as holding in both cases.

The eventual use case of the detector may often determine what combinations are needed from the originally detected target classes. For example, the use case in MAIJU-based analyses ideally needs filtering out the non-IM periods (no independent movement), because the MAIJU wearable is primarily used for the assessment of an infant's own motor performance^10,14^. We identified altogether five different binary definitions of interest to be used for automatic detection based on combinations of the annotated categories (Table 2; Fig. 1c), which range from a strict to loose criterion of what can be seen as “carrying” vs “non-carrying”.

**Table 2:** Candidate binary definitions for carrying/holding based on the annotated categories, alongside the human inter-rater agreement (kappa).

Binary definition	Non-carry categories	Carrying categories	Kappa
1 “AC versus rest”	IM, PS, AS, PC	AC	.74
2 “Carry versus rest”	IM, PS, AS	PC, AC	.77
3 “Active versus rest”	IM, PS, PC	AC, AC	.66
4 “IM + PS versus rest”	IM, PS	PS, AS, PC, AC	.76
5 “IM versus rest”	IM	AS, PC, AC	.85

### Annotation

Three trained human observers performed independent annotations using the open-source Anvil software (http://www.anvil-software.de/) that allows parallel visualization of the video and the sensor data. The annotators training consisted of an initial in-person training session, after which the annotators performed an exercise annotation of a 1-h recording, which was analyzed and reflected in another in-person session, where the potential questions that had arisen during the training annotation were addressed. Each recording received two independent annotations. In addition to the proposed categories, the annotators noted an extra category, “out of screen”, for time periods where the infant was not visible on the video. These time-periods were excluded from the loss function in classifier training.

### Neural network classifier

**Figure 3:**
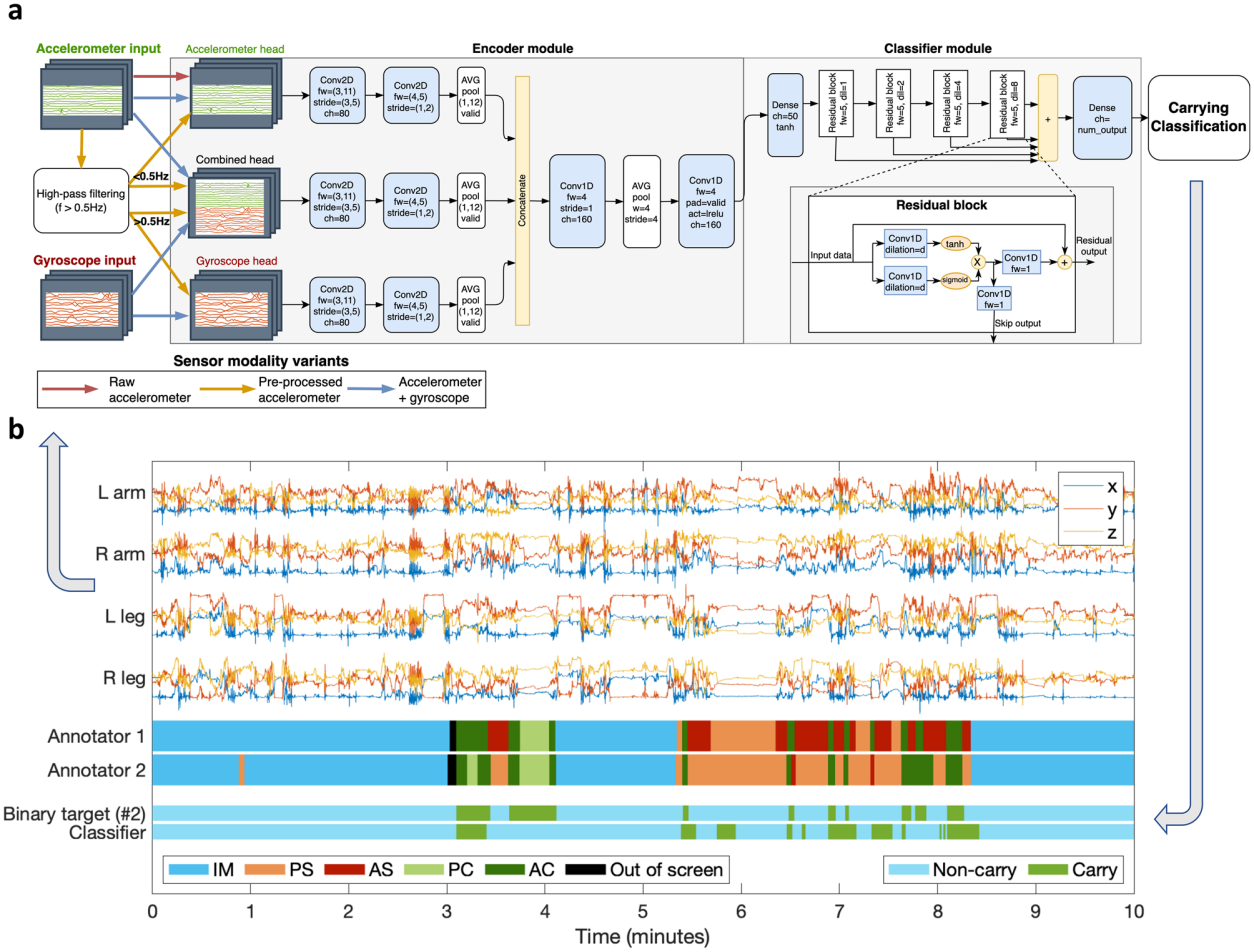
Classifier architecture and example recording. (**a**) The utilized end-to-end neural network classifier. The encoder module processes input data in modality-specific heads that are combined into a single latent representation vector. The classifier module models the time dynamics of the latent features to obtain the classification output. The input data pipelines for the sensor modality variants are color coded with red (“raw accelerometer”), orange (“pre-processed accelerometer”) and blue (“accelerometer + gyroscope”) arrows. (**b**) Example 10-min segment of a recording (accelerometer), annotations, and classifier output.

The neural network architecture (Fig. 3a,b) used in the present experiments was identical to that reported earlier^10,14^ consisting of an multi-head 2D-convolutional “encoder” part inspired by^24^ and a 1D-convolutional “timeseries modeling” part with residual connections and gated dilated convolutions inspired by^25^. In brief, the data is first segmented into 2.3 s (120 samples per channel) frames with 50% overlap (1.15 s; 60 samples). These frames are then fed as a batch sequence into the neural network classifier (Fig. 3a), where the encoder module performs frame-level (intra- and inter-) sensor fusion to obtain a 160-dimensional latent expression based on the raw accelerometer and gyroscope signals. The encoder takes in the accelerometer and gyroscope signals separately. In the case of accelerometer-only training, we tested two variants: First, a “raw” version, where the raw accelerometer data is input into the ‘accelerometer’ head, and the other encoder heads are ignored. Second, a “pre-processed” version, where low-pass filtered (*f* < 0.5 Hz) accelerometer data is fed into the accelerometer head and a high-pass filtered (*f* > 0.5 Hz) version of the accelerometer signal is used instead of the gyroscope signal. The high/low-pass filtering was used to split the gravity offset (containing orientational information) that is always present in the raw accelerometer signal from the rest (containing movement information). The high-pass filter was implemented as an 8th order Butterworth filter with a cut-off frequency of 0.5 Hz. The low-pass component was obtained by subtracting the high-pass component from the original signal. Finally, the classifier module models the frame-to-frame time dynamics of these features and outputs SoftMax probabilities for each target category.

All classifier training experiments were performed with tenfold cross validation with a fixed random seed. The training was performed with minibatch gradient descent using the ADAM algorithm (batch size 100 consecutive frames, learning rate 10^−4^, *β*_1_ = 0.9, *β*_2_ = 0.999, *ε* = 10^−8^) with a weighted categorical cross-entropy loss. For binary classifiers, a two-category output size was used. To mitigate the effects of unbalanced category distributions during training, each frame’s error in the loss function was weighted with the inverse probability of the target class occurrence. Sample and sensor dropout (both *p* = 0.3) were applied randomly to the input signals during training to ensure the robustness of the trained models. The training was run for 200 epochs and held out validation from the training split data (20% of training data) was used to select the best performing model in terms of the unweighted average F1 score.

### Effects of recording constellation on classifier performance

Next, we studied systematically how changes in the recording settings would affect the C/H classifier performance; in particular, we analyzed the effects of data sampling rate, sensor modality (accelerometer vs. gyroscope), or combinations of sensor placements. Sampling rate was studied with three different frequencies: 52 Hz (full resolution), 26 Hz, and 13 Hz. The low-resolution signals were obtained by decimating the full resolution signals by an order of 2 or 4, respectively, and then up-sampling and low-pass filtering the decimated signals back into the original resolution. The up sampling was performed to keep the classifier architectures identical. Only the signals from the selected sensors and modalities were concatenated into the classifier input. C/H definition #2 was used as the benchmark, and the null hypothesis (two-tailed paired *t*-test) was that the difference between the otherwise equivalent alternative recording settings is zero.

### Performance metrics

In the figures we use two main performance metrics for the results computed from the confusion matrix: Cohen’s kappa (*k*) and overall accuracy (acc). Thorough metrics, including precision, recall, and F1 score, are presented in Table 3.

1 $$\upkappa = \frac{{2 \cdot \left( {tp \cdot tn - fn \cdot fp} \right)}}{{\left( {tp + fp} \right) \cdot \left( {fp + tn} \right) + \left( {tp + fn} \right) \cdot \left( {fn + tn} \right)}}$$

2 $$acc = \frac{tp + tn}{{tp + tn + fp + fn}}$$

3 $$precision = \frac{tp}{{tp + fp}}$$

4 $$recall = \frac{tp}{{tp + fn}}$$

5 $$F1 = 2 \cdot \frac{precision \cdot recall}{{precision + recall}}$$

where *tp* = true positives, *tn* = true negatives, *fp* = false positives, *fn* = false negatives.

**Table 3:** Inter-rater agreements (Human vs. Human; left side) and classifier performances (Classifier vs. Human; right side) for the performed experiments (8 different classification tasks).

Raw annotations (5 class)		Human versus human		Accuracy: 91.9%		Classifier versus human		Accuracy: 80.3%	
Proportion annotated (%)		Kappa	Recall (%)	Precision (%)	F1 (%)	Kappa	Recall (%)	Precision (%)	F1 (%)
IM	86.8	0.85	99.5	96.6	98	0.24	83.8	96	89.5
PS	5.48	0.46	37.9	67.7	48.5	0.09	27.2	8.4	12.9
AS	2.43	0.32	61.3	23.2	33.7	0.06	19.2	4.8	7.6
PC	2	0.45	31.5	84.8	46	0.11	14.9	9.7	11.7
AC	3.31	0.75	76.9	73.8	75.3	0.44	63	35.5	45.4
W Average		0.67	91.9	92.5	91.4	0.21	80.3	90.2	84.6
UW Average			61.4	69.2	60.3		41.6	30.9	33.4

IM versus passive versus active (3 class)		Human versus human		Accuracy: 93.4%		Classifier versus human		Accuracy: 84.2%	
Proportion annotated (%)		Kappa	Recall (%)	Precision (%)	F1 (%)	Kappa	Recall (%)	Precision (%)	F1 (%)
IM	86.8	0.85	99.5	96.6	98	0.29	87.3	96.2	91.5
Passive	7.5	0.46	45.3	89.7	60.2	0.14	30.8	13.4	18.7
Active	5.7	0.32	84.8	56.3	67.6	0.39	61.8	31.7	41.9
W Average		0.73	93.4	94	92.8	0.27	84.2	90.6	86.9
UW Average			76.5	80.8	75.3		60	47.1	50.7

IM versus support versus carry (3 class)		Human versus human		Accuracy: 94.4%		Classifier versus human		Accuracy: 83.7%	
Proportion annotated (%)		Kappa	Recall (%)	Precision (%)	F1 (%)	Kappa	Recall (%)	Precision (%)	F1 (%)
IM	86.8	0.85	99.5	96.6	98	0.3	86.6	96.4	91.2
Support	7.9	0.63	62.2	69.8	65.8	0.11	30.8	10.7	15.9
Carrying	5.3	0.77	67.5	93.1	78.2	0.47	65.4	39.9	49.6
W Average		0.77	94.4	94.1	94.1	0.28	83.7	91.1	86.8
UW Average			76.4	86.5	80.7		60.9	49	52.2

Def #1 (binary)		Human versus human		Accuracy: 98.4%		Classifier versus human		Accuracy: 96.7%	
Proportion annotated (%)		Kappa	Recall (%)	Precision (%)	F1 (%)	Kappa	Recall (%)	Precision (%)	F1 (%)
IM+PS+AS+P	96.7	0.75	99.1	99.2	99.2	0.5	97.5	99	98.3
AC	3.3	0.75	76.9	73.8	75.3	0.5	65.4	42.5	51.5
W Average		0.75	98.4	98.4	98.4	0.5	96.7	97.5	97
UW Average			88	86.5	87.2		81.5	70.8	74.9

Def #2 (binary)		Human versus human		Accuracy: 97.7%		Classifier versus human		Accuracy: 96.3%	
Proportion annotated (%)		Kappa	Recall (%)	Precision (%)	F1 (%)	Kappa	Recall (%)	Precision (%)	F1 (%)
IM+PS+AS	94.7	0.77	99.7	97.9	98.8	0.56	97.4	98.8	98.1
PC+AC	5.3	0.77	67.5	93.1	78.2	0.56	68.5	50.5	58.2
W Average		0.77	97.7	97.6	97.5	0.56	96.3	97	96.6
UW Average			83.6	95.5	88.5		83	74.7	78.1

Def #3 (binary)		Human versus human		Accuracy: 96.3%		Classifier versus human		Accuracy: 93.9%	
Proportion annotated (%)		Kappa	Recall (%)	Precision (%)	F1 (%)	Kappa	Recall (%)	Precision (%)	F1 (%)
IM+PS+PC	94.3	0.66	96.8	99.3	98	0.4	95.1	98.6	96.8
AS+AC	5.7	0.66	84.8	56.3	67.7	0.4	63.2	32.3	42.8
W Average		0.66	96.3	97.3	96.6	0.4	93.9	96.2	94.8
UW Average			90.8	77.8	92.8		79.1	65.4	69.8

Def #4 (binary)		Human versus human		Accuracy: 96.6%		Classifier versus human		Accuracy: 93.7%	
Proportion annotated (%)		Kappa	Recall (%)	Precision (%)	F1 (%)	Kappa	Recall (%)	Precision (%)	F1 (%)
IM+PS	92.3	0.76	97.9	98.4	98.1	0.47	94.9	98.4	96.6
AS+PC+AC	7.7	0.76	80.3	75.4	77.8	0.47	67.5	39.2	49.6
W Average		0.76	96.6	96.7	96.6	0.47	93.7	95.7	94.5
UW Average			89.1	86.9	88		81.2	68.8	73.1

Def #5 (binary)		Human versus human		Accuracy: 96.5%		Classifier versus human		Accuracy: 86.6%	
Proportion annotated (%)		Kappa	Recall (%)	Precision (%)	F1 (%)	Kappa	Recall (%)	Precision (%)	F1 (%)
IM	86.8	0.85	99.5	96.6	98	0.32	89	96.2	92.5
PS+AS+PC+AC	13.2	0.85	79.1	96.4	86.9	0.32	56.7	29.5	38.8
W Average		0.85	96.5	96.5	96.4	0.32	86.6	91.2	88.5
UW Average			89.3	96.5	92.4		72.8	62.9	65.6

Metrics include Accuracy, kappa, recall, precision, and F1-score. The weighted scores are based on the annotated proportions.

For classifier performance, the confusion matrix is computed based on the frames where the human annotators were in agreement. In all cases, the frames denoted as “out of screen” in the annotations were discarded. Overall accuracy answers to the question “what is the probability that a random frame from a recording is classified correctly”. Due to the unbalanced nature of the category distributions, however, its information value is limited in assessing classifier performance in less represented categories. To balance this, we use the kappa score which gives a balanced correlation-like score from [− 1, 1], where a score of 0 denotes chance-level performance (1 being perfect and -1 anti-perfect). It could be argued that the similar Matthew’s Correlation Coefficient (MCC) would be a superior metric to kappa, especially for imbalanced category distributions^26^, but we chose to use kappa due to its conventional position in reporting inter-rater agreement.

### Comparison to actigraphy-based detection

Actigraphy refers to the common methodology of using single wrist- or ankle-placed accelerometers to measure the overall quantity of movements over time. It is commonly used to quantify physical activity as a cumulative count of active and/or inactive epochs in a large variety of contexts, such as developmental studies^15,27^ or sleep studies^28^. The exact algorithms used in actigraphy analyses may be undisclosed or they vary substantially, with the most common directions being (1) digital integration of signal energy, (2) zero-crossing rate, and (3) time above a threshold^28^. For the present study, we studied how well the thresholding of digital integration-based features can perform in the classification tasks #1–5. The algorithm was implemented similarly to^29^, where the magnitude signal from the tri-axial accelerometer signals *a*_*x,y,z*_ was obtained as

3 $${a}_{mag}=\sqrt{{a}_{x}^{2}+{a}_{y}^{2}+{a}_{z}^{2}}$$

The resulting actigraphy signal (*a*_*mag*_) was band-pass filtered into frequency range of 1–6 Hz with a 4th-order Butterworth filter, and a moving integral value was computed for each 2.3-s frame as the sum of absolute magnitude values within the frame.

### Ethics approval and consent to participate

The study was approved by the relevant Ethics Commission and the Children’s Hospital, Helsinki University Hospital, Helsinki, Finland. All methods were performed in accordance with the relevant guidelines and regulations in place in Children’s Hospital of Helsinki University Hospital. An informed consent was obtained from the legal guardian(s) of all subjects. An informed written consent was also obtained from the legal guardian(s) of the infant shown in Fig. 1a to be used in an online open-access publication.

## Results

### E1: Annotation

#### Distributions

The recording-level statistics for the annotations are shown in Fig. 2b for both annotators. The overall statistics are presented in Table 3. Independent movement was the dominant category, with an overall mean value of 86% (range 64–97%) annotated. The next most common category was “support passive” with 5.6% (0–17%), followed by “active carrying” 3.5% (0.2–13%), “active support” 2.5% (0–13%), and “passive carrying” 2.1% (0–15%).

#### Segment length analysis

The cumulative distributions of annotated segment lengths for the raw annotations (Fig. 2c) and binary definitions (Fig. 2d) show that over 40% of annotations have a segment length between 1 and 5 s, corresponding to roughly 10% of the total amount of annotated C/H time. For the binary definitions, the annotated segment lengths get longer (i.e., the cumulative distribution becomes flatter) with looser definitions, as expected. These distributions suggest suitable lower bounds for the classifier’s temporal resolution between 1 and 5 s, because a large proportion (~ 40%) of individual segments are within this range. The optimal window length is a trade-off between phenomenal resolution (given by the annotations) and the amount of recording data to base the classification on (given by the sensors). To harmonize analytic pipelines, we found it reasonable to use window length of 2.3 s with 50% overlap for the present C/H detection, because the same was already used in our previous classifier development for the MAIJU-based motor assessment^10^ (Fig. 2c,d).

#### Human inter-rater agreement

**Figure 4:**
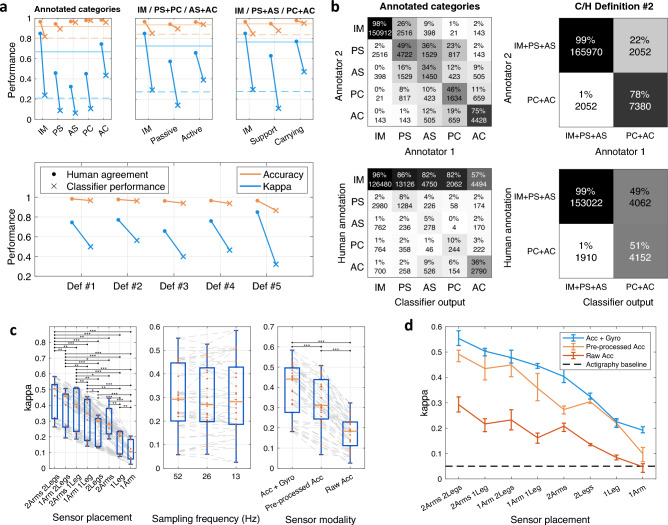
Human inter-rater agreement and classifier performance. (**a**) Human inter-rater agreement and classifier performance metrics for the definitions of C/H or physical infant-parent interaction. Results are reported as accuracy and Cohen’s kappa. For the top row, the horizontal lines denote the overall inter-rater agreement (solid) and the classifier vs human performance (dashed). (**b**) Confusion matrices for the human inter-rater agreement (top) and human vs machine classifications (bottom) for the original annotations (left) and the best-performing binary definition, C/H Definition 2 (right). (**c**) Effect of sensor placement (left), sampling frequency (middle), and sensor modality (right) on the classification performance (kappa) for C/H Definition 2. The boxplots show the median, IQR and range of experiment values for the cases where the given attribute is held constant. The gray lines connect systems with otherwise equivalent parameters. Two-tailed paired *t*-tests are used to test for the null hypothesis that the differences between otherwise equivalent systems’ performances are zero (*p < .05; **p < .005; ***p < .0001). (**d**) Summary of the findings presented in (**c**), with sensor modalities indicated by different colors, and the range of the sampling frequencies is plotted with whiskers. Note the systematic decline in classifier performance when reducing the sensor combination from the four-limb recordings to single-sensor recording from the arm. Also note the superiority of classification based on a combination of accelerometer and gyroscope (blue), as well as the markedly poorer classification performance when using the raw accelerometer signals only (orange and red).

The inter-rater agreement for the full five-category annotation was *k* = 0.67 (Cohen’s kappa). For single-category/binary classifications, the inter-rater agreements ranged from 0.32 to 0.85 (Tables 1, 2 and 3, Fig. 4a). IM and AC have very high agreement levels, but the other categories (PS, AS, PC) have considerably lower agreement between 0.32 and 0.46. The confusion matrices of the original annotations (Fig. 4b, top left) show that AS is the most ambiguous category, with a considerable confusion with all the other categories. However, combining the original annotations into three-category variants (IM vs. Passive vs. Active or IM vs. Support vs. Carry; Fig. 4a top middle and top right; Table 3) leads to a considerable increase in the inter-rater agreement (*k* = 0.72 and 0.76, respectively). For the binary definitions, the inter-rater agreement is around *k* = 0.76 for C/H definitions #1, #2, and #4, and the lowest (0.56) for #3. Definition #5, corresponding to IM, scores the highest kappa (0.85).

### E2: Classification performance

The feasibility of classifier training was tested for the full categorization scheme (Fig. 1b) by training classifiers with the full 4-sensor MAIJU data (both accelerometer and gyroscope at 52 Hz). The C/H definition with the best-performing classifier was then selected for further experiments where we studied the effect of sensor placement, sampling rate, and sensor modality (accelerometer vs gyroscope) to classifier performance.

#### Carrying definitions

The performance of the classifier algorithm is detailed in Fig. 4a and Table 3 alongside the corresponding human inter-rater agreement metric. We first examined the full multi-class classifier (top row): it was found to perform at an overall level of *k* = 0.2–0.3, and the category-specific kappa values for these classifiers is within the range 0.1–0.5. Despite being above chance level, the performance is too low to be of practical utility; that is clearly seen in the confusion matrix for the original five-category classifier (Fig. 4b, bottom left), where the classifier output is heavily biased towards the IM category in every case. For the single categories, the best-performing metrics are obtained with AC (for original categories; *k* = 0.43) and “Carrying” (AC + PC, *k* = 0.47) in the IM vs Support vs Carrying classifier. It is important to note, however, that IMU sensor-based classification performance does not necessarily follow the video-based inter-rater agreement of the annotations, as is well seen in the performance of IM (*k*_*IM,irr*_ = 0.85 to *k*_*IM,cls*_ = 0.24).

Next, we examined how much classifier performance could be improved by re-defining the target categories into a binary classification task (Fig. 4a, bottom), which resulted in a far higher performance: The best performing C/H binary definitions were #2 (AC + PC, *acc* = 96%, *k* = 0.56) and #1 (AC, *acc* = 97%, *k* = 0.50). Intriguingly, the confusion matrix for #2 (Fig. 4b, bottom right) shows that the type I error (false positives) for carrying is on a par with the inter-rater agreement confusion matrix, while type II error (false negatives) is inferior to the annotations, missing roughly half of the 2.3 s frames of carrying.

#### Effects of recording constellation on classifier performance

The results are detailed in Fig. 4c and d. Figure 4c presents performance (kappa) of all classifier alternatives that were trained with the common property fixed. The greatest effect on classifier performance was found to be caused by the sensor placement. The full four-sensor recording yielded the best result (*t*(8) > 4, *p* < 0.005 for all). The three-sensor recordings (1 arm + 2 legs vs. 2 arms + 1 leg) were somewhat inferior to the four-sensor recordings, but they showed no significant differences between each other (*t*(< 8) = 0.07, *p* = 0.9) . For two-sensor recordings, the best classifier performance was obtained with a recording including one arm and one leg, which was significantly better than the recordings including both legs (*t*(8) = 3.8 , *p* < 0.05). For single-sensors, one leg was significantly better than one arm (*t*(8) = 3.8, *p* < 0.005), but both of them showed a very poor overall performance (*k* range 0.02–0.25).

There were no significant differences (*t*(23) range = 0.02–0.5, *p* > 0.05) between the sampling rate variants; therefore, the Fig. 4c data is presented in an alternative way in Fig. 4d, showing each sensor modality as an own track according to sensor placements, and the results with different sampling rates are presented with the range.

The effect of sensor modality was statistically very significant (*t*(23) range = 5.6–18, *p* < 0.0001). However, the difference between recordings with and without gyroscope was only modest when the accelerometer data was pre-processed by splitting the low-pass gravitational component from the high-pass part. The classifier based on the “raw accelerometer” data yielded a clearly lower performance (*k* = 0.3).

These experiments together indicate that C/H classification is significantly improved by adding more sensors, and the performance could perhaps improve even further with more than four sensors. Sensor locations did also contribute to the classifier performance, especially when both upper and lower extremities were included. Sampling rate, however, was not found to affect the results in a meaningful manner. These findings have practical implications since higher data rates will pose challenges to a continuous data streaming over the BLE connection, and in many cases a high number of sensors may be otherwise impractical. The practical compromise between classifier performance and minimal recording configuration was found to be a combination of two sensors (1 arm + 1 leg), using both accelerometer and gyroscope, and 13 Hz sampling rate, yielding *k* = 0.45.

### Comparison to actigraphy-based detection

**Figure 5:**
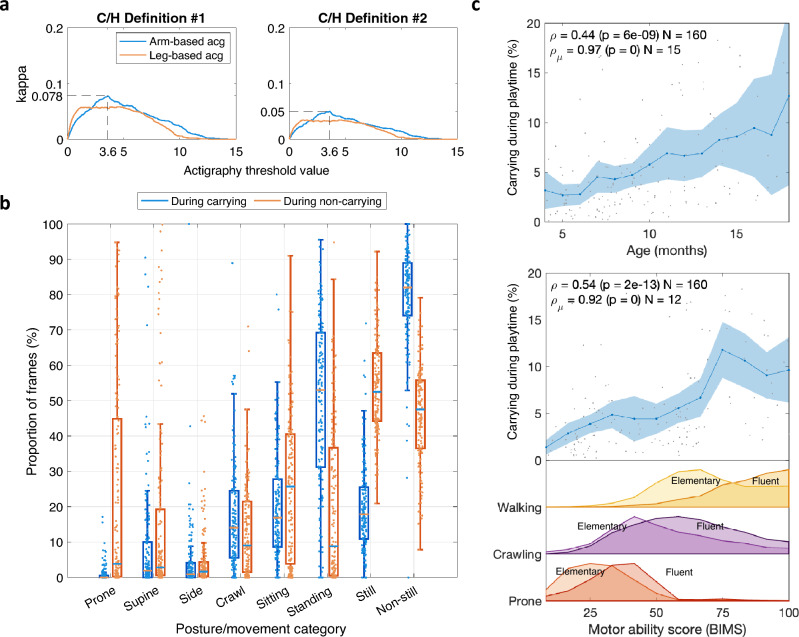
Proof-of-concept application in developmental research. (**a**) Actigraphy-based classification performance as a function of threshold value for C/H definition #1 (left; best-performing with actigraphy) and #2 (right; best-performing with proposed classifier). Optimal performance points shown with dashed lines. (**b**) Infant postures and movements during detected carrying (blue) and non-carrying (orange) within free play time. Box plots show the median, IQR and range of the data (N = 162). (**c**) Proportion of detected carrying from periods of free play time as a function of infant age (top) and motor ability score (bottom). Group average trajectories are shown with blue lines alongside their 95% confidence intervals (blue areas). Spearman’s ρ reported for the individual recordings (N = 162) and for the group-average trajectory (ρ_μ_). The average amount of detected prone crawling (red), crawling (purple), and walking (yellow) are shown as a function of the BIMS motility score, split into elementary (light) and fluent (dark) qualitative descriptors.

The results obtained with varying thresholds for the actigraphy-based detection for C/H definitions #1 (best-performing for actigraphy) and #2 (best performing for proposed method) are presented in Fig. 5a. The classification performance was very poor (*k* < 0.1 for all C/H binary definitions), indicating a nearly chance-level classification. Moreover, the finding was essentially unaffected by increasing the window lengths from 1.15 to 10 s. These results approach the lower-end in the variation seen with the properly trained classifier using data from a single arm-attached accelerometer sensor; but the actigraphy results were considerably worse than the properly trained classifier based on single-sensor data from one leg (Fig. 4d; *k*_*acg*_ = 0.06 vs. *k*_*proposed*_ = 0.25).

### Proof-of-concept application in developmental research

#### Infant postures during carrying

In our final set of experiments, we showcase the potential utility of the trained carrying classifier alongside the MAIJU wearable in studying physical infant-caregiver interactions. For these examples we utilize another dataset (DS-2) of minimally supervised at-home recordings (without video) consisting of *N* = 160 recordings from 58 infants (age range 4–18 months)^23^. The recordings were processed and automatically classified for carrying with the proposed method (C/H definition #2), as well as concurrently classified for posture and movement based on the MAIJU analysis pipeline^10,23^. Only those time windows were retained from the multi-hour recordings that were indicated by the parents as “free play time”.

The C/H classification may be informative per se, but adding the concurrent posture and movement information from the MAIJU pipeline facilitates a more detailed analysis of the C/H behavior. Figure 5B depicts the MAIJU-based distributions of postures and movement/non-movement during detected carrying time periods (blue) and during non-carrying time periods (orange). The findings suggest that standing is the most common posture for carrying infants during our recordings, followed by sitting and crawl postures. The distributions for movement, still vs non-still, show a wider variability during carrying, which is clearly dominated by the non-still epochs. There are clear differences in movement distributions during non-carrying and carrying, suggesting removal of carrying epochs would improve the estimates of independent infant motility. Conversely, the information about carrying postures and activity could be used to design research questions regarding the quality of physical infant-caregiver interactions.

#### Amount of carrying as a function of motor development

Figure 5c (top) depicts the average amount of carrying during the play times as a function of infant age between 4 and 18 months, alongside the average occurrence of the three major movement modalities (prone crawl, crawling, walking) split into qualitative “elementary” and “fluent” descriptors^10^. The amount of carrying increases with age (Spearman’s *ρ* = 0.44, *p* = 10^−10^; average trend *ρ* = 0.97, *p* = 0). This may be already interesting in itself; however the present results allow even more fine-grained analysis: the child’s ages can be converted to estimates of motor ability at the time of the recording (i.e., BABA Infant Motor Score, BIMS, the age prediction based on the posture and movement distributions, described in^10^**)**. As shown in Fig. 5c (bottom), the population-level correlation increases to *ρ* = 0.54 (*p* = 10^−12^) and two humps in the incidence of carrying emerge at around 30 and 75 points of the BIMS score, respectively. Comparison to the movement distributions show that these humps coincide with the newly emerged fluent movement patterns, crawling and walking, respectively. An intuitively appealing *post-hoc* explanation for this observation could be that the novel movement abilities trigger increased attention and carrying behavior from the caregivers to ensure infants’ safety as their sphere of activity increases (e.g., keeping out of harm’s way, picking, and consoling after falling).

## Discussion

Our study shows that automatic C/H detection is feasible from the recordings collected at home during normal daily activities. Expectedly, the accuracy of such detection depends very much on the phenomenological classification of carrying/holding, and the accuracy follows the manner how the given C/H definition manifests in the movements of the infant trunk and extremities. The presently described algorithm allows analyses from data recorded in a fully unsupervised manner in various out-of-hospital/lab environments. Our work shows further that an actigraphy-type single sensor recording cannot provide C/H detections at fine-grained timescales, which constitute over 40% of the annotated carrying epochs in our annotated dataset. Finally, we show a proof-of-concept example in the context of tracking early motor development, where parental C/H behavior can be matched with individual level motor performance of the given infant.

Detection and classification of behavioral repertoires, such as carrying and holding, is challenged by the inherent ambiguities in the phenomenological categories. Here, we developed a systematic approach to categorize C/H in ways that can be measured using movement sensors (see Methods). Our results report a systematic characterization of the algorithm’s performance for those newly categorized C/H behaviors, which is believed to support further use of this algorithm, or development of other algorithms. The eventual use case of the C/H detector may determine what category combinations are needed from the originally detected target classes. For example, the use case in MAIJU-based analyses ideally needs filtering out the non-IM periods (no independent movement), because MAIJU wearable is primarily used for assessing an infant's own motor performance^10^. However, our results show that IMU-based recordings are not effective in discriminating based on this category.

Our experiments together indicate that C/H classification is significantly improved by adding more sensors, and the performance could perhaps improve even further with more than four sensors. Sensor locations did also contribute to the classifier performance, especially when both upper and lower extremities were included. Sampling rate, however, was not found to affect the results in a meaningful manner. These findings have practical implications since higher data rates will pose challenges to a continuous data streaming over the BLE connection, and in many cases a high number of sensors may be otherwise impractical. The practical compromise between classifier performance and minimal recording configuration was found to be a combination with two sensors (1 arm + 1 leg), using gyroscope and 13 Hz sampling rate, yielding *k* = 0.45. It is important to note, however, that C/H detection from IMU sensors requires sensed movements (from the caregiver and/or infant) during the target behavior. Other types of C/H behaviors^30^ could be recorded with other instrumentation, such as proximity sensors^6,31^ or video recordings, both of which have their own practical and technical limitations. A particular advantage of the IMU sensors is that the same data can be used for measuring other features of infants’ activity, such as quantification of play time, posture, movement, as well as a comprehensive assessment of motor abilities^10^.

A key advantage of the novel algorithm is the ability to provide C/H detections for long term recordings from fully unsupervised settings, which allows monitoring of normal daily activities^1^. Technically, the algorithm was shown to be robust for a range of sampling frequencies and sensor combinations, implying flexibility in instrumentations. These recordings can be conveniently performed using the MAIJU wearable that is openly available, scalable, and well accepted by the researchers and caregivers^10,14^. Unlike many other solutions, recordings with the MAIJU wearable do not require additional sensors attached to the environment or the caregivers (cf.^13,32^) making it easier to generalize in different study scenarios.

A direct comparison of our present results to prior studies is difficult for several reasons: First, the present work is, to our knowledge, the first one to develop C/H detection at a high temporal resolution for data collected at home in an unsupervised manner. Prior studies have used lower temporal resolution and instructed behaviors^13^ which would automatically lead to higher detection performance; however, such approaches violate the ecological validity by dismissing the very frequently occurring brief episodes of holding and carrying, as was shown in our analysis of video-based annotations. Second, prior studies have not presented a systematic phenomenological scheme akin to our C/H categories, hence their classification tasks per se may not be fully comparable to ours. Nevertheless, it is worth noting that our best-performing C/H binary definition #2 is likely close to infant holding as defined by^13^. Our present results are compatible with their findings in that IMU sensors can be used for detecting such C/H behavior. The numerical performance measures from the present study are not directly comparable to^13^ due to marked differences in the dataset collection (supervised actions in their study vs. spontaneous daily activity in our study). Using a supervised (i.e., acted) recording setting in^13^ compromises ecological validity of the findings, and it directly biases the category distributions, both of which would lead to a higher formal detector performance. Third, prior studies with at least a decent classifier performance were based on a multi-person instrumentation where sensors are attached to both the parents and infants^13,32,33^; in contrast, our study was based on infant-worn sensors only. Sensing from multiple persons will undoubtedly bring technical accuracy to detecting proximity and co-incident movements with the caregiver, however such instrumentation also brings in significant complexity in the study logistics.

There are some limitations that need to be considered when applying our C/H detector in prospective studies. First, the algorithm is trained and tested only with data obtained from “play sessions”, which likely represents ecologically relevant and diverse behaviors; however, the play sessions do not include all the C/H behavior variants that may occur within a day-long cycle (e.g., diaper change, feeding). Second, the scaling/post-processing of the proposed method to lower temporal resolutions (e.g., > 15 s) to obtain more robust estimates for aggregated C/H time was not within the scope of the current study. We are however confident that the fine-grained classifier output could be readily used as the basis features for this task. Third, the algorithm is trained for the sensor locations that are used in the MAIJU wearable, thus alternative sensor configurations do need a new training. Fourth, the C/H detector performance is physically limited by the abilities of movement sensors to discriminate between different types in the spectra of C/H behaviors. For instance, movement-based C/H detection is not able to recognize a moment when the caregiver is holding a still infant. Such a situation could be indirectly estimated from the body posture, as provided by the postural detection in our MAIJU analysis pipeline^10^, or directly measured using proximity sensors^6,31^, which require additional sensors attached on the respective caregiver^6,13^. Fifth, it is also important to recognize the phenomenological ambiguities in C/H behavior; for instance, a child climbing on a still caregiver can be considered as moving independently or as being held by the caregiver. Such ambiguities cannot be resolved with algorithmic solutions, but they call for question-guided instrumentation coupled with a careful consideration of the C/H phenomenology.

An automated and objective algorithm for C/H detection can be used in multiple ways: First, the C/H detector could be used as a component in a larger analysis pipeline. Our present work was initially motivated by the need to improve the specificity of motor assessment with the MAIJU wearable^10^, which may be readily confounded by carrying or holding the infant during unsupervised measurement sessions. We reasoned that using C/H detection in the preprocessing phase of the automated MAIJU analysis pipeline would improve focusing on time periods when the infants are moving by themselves. The same need to distinguish a child’s own activity from other physical movements is clear in studies that aim to understand developmental origins of childhood obesity and related health adversities^34^. Second, there are also many other comparable needs in infant behavioral research where unsupervised measurement sessions need to be segmented for movement periods by the infant versus by an external force^13,16^. For instance, studies on parent-infant interaction or child’s language development in natural environments may utilize audio signals^35,36^; the yield of such data analyses could be substantially improved by segmenting and assessing the results with respect to C/H epochs. Third, the C/H detector could be particularly useful when used in combination with other algorithmic assessments of infants’ performance; taken together they may provide a “multimodal behavioral assessment” by using the same physical recording data. Our proof-of-context experiment shows how C/H behavior can be quantified in the context of “play time” recognized by a third algorithm. This algorithm combination disclosed an age-related increase in C/H across the age range in our population; moreover the results suggested that C/H may relate to the individual-level transition from predominantly crawling to walking activity. It is intuitively conceivable that infants’ attempts to move in a novel manner are reflected in caregivers’ C/H behavior. Such causal relations will obviously remain only speculative with this retrospectively analyzed data, yet the observations per se provide a clear case for the potential of our C/H detection algorithm. Fourth, C/H detection may be useful for understanding the relationships between infants’ motor activity and social or neurocognitive development^37^. Infants’ own motor activity is considered to facilitate exploratory behavior, which in turn is essential for neurocognitive development^38^. Studying these relationships would benefit from methods that can distinguish parental C/H behavior from self-initiated movements.

## Data Availability

The data or materials for the experiments reported here can be made available at reasonable request and within relevant legal constraints. Please contact Sampsa Vanhatalo (sampsa.vanhatalo@helsinki.fi) for requests.

## References

[1] de Barbaro K (2019). Automated sensing of daily activity: A new lens into development. Dev. Psychobiol..

[2] Health, G. B. P. H. of C. & Committee, S. C. *First 1000 Days of Life: Thirteenth Report of Session 2017–19. Report*. https://publications.parliament.uk/pa/cm201719/cmselect/cmhealth/1496/1496.pdf (2019).

[3] Mendoza JK, Fausey CM (2021). Quantifying everyday ecologies: Principles for manual annotation of many hours of infants’ lives. Front. Psychol..

[4] Walker SP (2007). Child development: Risk factors for adverse outcomes in developing countries. The Lancet.

[5] Franchak JM, Scott V, Luo C (2021). A contactless method for measuring full-day, naturalistic motor behavior using wearable inertial sensors. Front. Psychol..

[6] Salo VC (2022). Measuring naturalistic proximity as a window into caregiver–child interaction patterns. Behav. Res. Methods.

[7] Cychosz M (2020). Longform recordings of everyday life: Ethics for best practices. Behav. Res. Methods.

[8] Fish LA, Jones EJH (2021). A survey on the attitudes of parents with young children on in-home monitoring technologies and study designs for infant research. PLOS ONE.

[9] Levin HI (2021). Sensing everyday activity: Parent perceptions and feasibility. Infant Behav. Dev..

[10] Airaksinen M (2022). Intelligent wearable allows out-of-the-lab tracking of developing motor abilities in infants. Commun. Med..

[11] Wilson RB, Vangala S, Elashoff D, Safari T, Smith BA (2021). Using wearable sensor technology to measure motion complexity in infants at high familial risk for autism spectrum disorder. Sensors.

[12] Abrishami MS (2019). Identification of developmental delay in infants using wearable sensors: Full-day leg movement statistical feature analysis. IEEE J. Transl. Eng. Health Med..

[13] Yao X, Plötz T, Johnson M, de Barbaro K (2019). Automated detection of infant holding using wearable sensing: Implications for developmental science and intervention. Proc ACM Interact Mob Wearable Ubiquitous Technol.

[14] Airaksinen M (2020). Automatic posture and movement tracking of infants with wearable movement sensors. Sci. Rep..

[15] Bruijns BA, Truelove S, Johnson AM, Gilliland J, Tucker P (2020). Infants’ and toddlers’ physical activity and sedentary time as measured by accelerometry: A systematic review and meta-analysis. Int. J. Behav. Nutr. Phys. Act..

[16] Jun K, Choi S (2020). Unsupervised end-to-end deep model for newborn and infant activity recognition. Sensors.

[17] Worobey J, Vetrini NR, Rozo EM (2009). Mechanical measurement of infant activity: A cautionary note. Infant Behav. Dev..

[18] Yoshida S, Funato H (2021). Physical contact in parent-infant relationship and its effect on fostering a feeling of safety. iScience.

[19] Anisfeld E, Casper V, Nozyce M, Cunningham N (1990). Does infant carrying promote attachment? An experimental study of the effects of increased physical contact on the development of attachment. Child Dev..

[20] Ressman J, Grooten WJA, Rasmussen Barr E (2019). Visual assessment of movement quality in the single leg squat test: A review and meta-analysis of inter-rater and intrarater reliability. BMJ Open Sport Amp Exerc. Med..

[21] Wittek N, Wittek K, Keibel C, Güntürkün O (2022). Supervised machine learning aided behavior classification in pigeons. Behav. Res. Methods.

[22] Stevenson NJ (2015). Interobserver agreement for neonatal seizure detection using multichannel EEG. Ann. Clin. Transl. Neurol..

[23] Airaksinen, M. *et al.* Charting infants’ motor development at home using a wearable system: Validation and comparison to physical growth charts. *BioMedicine* **92** (2023). 10.1016/j.ebiom.2023.104591 PMC10176156 37137181

[24] Ha, S. & Choi, S. Convolutional neural networks for human activity recognition using multiple accelerometer and gyroscope sensors. In *2016 International Joint Conference on Neural Networks (IJCNN)* 381–388 (2016). 10.1109/IJCNN.2016.7727224.

[25] Oord, A. van den *et al.* WaveNet: A generative model for raw audio. arXiv:1609.03499 (2016).

[26] Chicco D, Warrens MJ, Jurman G (2021). The Matthews correlation coefficient (MCC) is more informative than Cohen’s kappa and brier score in binary classification assessment. IEEE Access.

[27] Hoyt CR (2019). Detection of pediatric upper extremity motor activity and deficits with accelerometry. JAMA Netw. Open.

[28] Jean-Louis G, Kripke DF, Mason WJ, Elliott JA, Youngstedt SD (2001). Sleep estimation from wrist movement quantified by different actigraphic modalities. J. Neurosci. Methods.

[29] Ranta J (2021). An openly available wearable, a diaper cover, monitors infant’s respiration and position during rest and sleep. Acta Paediatr..

[30] Bigelow AE, Williams LR (2020). To have and to hold: Effects of physical contact on infants and their caregivers. Infant Behav. Dev..

[31] Ozella L (2018). Close encounters between infants and household members measured through wearable proximity sensors. PLOS ONE.

[32] Patel P, Shi Y, Hajiaghajani F, Biswas S, Lee M-H (2019). A novel two-body sensor system to study spontaneous movements in infants during caregiver physical contact. Infant Behav. Dev..

[33] Fujii S, Watanabe H, Taga G (2020). Wearable strain sensor suit for infants to measure limb movements under interaction with caregiver. Infant Behav. Dev..

[34] Eichner-Seitz N, Pate RR, Paul IM (2023). Physical activity in infancy and early childhood: A narrative review of interventions for prevention of obesity and associated health outcomes. Front. Endocrinol..

[35] Wang Y (2017). A systematic review of the use of LENA technology. Am. Ann. Deaf.

[36] Ganek H, Eriks-Brophy A (2018). Language ENvironment analysis (LENA) system investigation of day long recordings in children: A literature review. J. Commun. Disord..

[37] Franchak JM (2019). Changing opportunities for learning in everyday life: Infant body position over the first year. Infancy.

[38] Hoch JE, O’Grady SM, Adolph KE (2019). It’s the journey, not the destination: Locomotor exploration in infants. Dev. Sci..

